# Real-World Effectiveness of Nirmatrelvir–Ritonavir Against Severe Outcomes of COVID-19 in Taiwan: A Nationwide Population-Based Cohort Study

**DOI:** 10.1093/ofid/ofaf553

**Published:** 2025-09-02

**Authors:** Raymond N Kuo, Wanchi Chen, Wen-Yi Shau, Shan-Chwen Chang

**Affiliations:** Institute of Health Policy and Management, College of Public Health, National Taiwan University, Taipei City, Taiwan; Population Health Research Center, National Taiwan University, Taipei City, Taiwan; Institute of Health Policy and Management, College of Public Health, National Taiwan University, Taipei City, Taiwan; Graduate Institute of Clinical Medicine, College of Medicine, National Taiwan University, Taipei City, Taiwan; Graduate Institute of Clinical Pharmacy, College of Medicine, National Taiwan University, Taipei City, Taiwan; Department of Internal Medicine, National Taiwan University Hospital, Taipei City, Taiwan; Department of Internal Medicine, College of Medicine, National Taiwan University, Taipei City, Taiwan

**Keywords:** COVID-19, effectiveness, nirmatrelvir–ritonavir, severe outcomes, Taiwan

## Abstract

**Background:**

Real-world data evaluating the effectiveness of nirmatrelvir–ritonavir across diverse age groups and vaccination statuses remain limited, particularly in East Asian populations. This study evaluates its effectiveness in reducing severe COVID-19 outcomes using a large, comprehensive nationwide healthcare database and to provide new evidence.

**Method:**

This retrospective observational cohort study involved outpatient COVID-19 patients diagnosed between 1 January 2022, and 1 December 2022, within Taiwan's National Health Insurance Research Database. Patients who received nirmatrelvir–ritonavir were compared with untreated patients. Primary outcomes include COVID-19-related hospitalization, ICU admission, invasive ventilatory support, death, and the composite outcome of hospital admission or death.

**Results:**

A total of 2 300 131 nonhospitalized patients with confirmed COVID-19 between 1 January 2022 and 1 December 2022, including 530 807 patients treated with nirmatrelvir–ritonavir and 1 769 324 untreated patients. Treatment with nirmatrelvir–ritonavir was associated with a significantly lower risk of COVID-19-related hospitalization (hazard ratio 0.32 [95% CI .31–.34]), ICU admission (0.41 [.38–.45]), invasive ventilatory support (0.38 [.33–.43]), death (0.42 [.40–.45]), and the composite outcome of hospital admission or death (0.34 [.33–.35]). Effectiveness was consistent across subgroups stratified by age and vaccination status, with the greatest benefit observed in unvaccinated individuals and those aged ≥65 years with additional risk factors.

**Conclusions:**

In a large, nationally representative cohort, outpatient use of nirmatrelvir–ritonavir was associated with a significantly lower risk of severe COVID-19-related outcomes, regardless of age or vaccination status. These findings reinforce the clinical value of early antiviral treatment, particularly in aging and unvaccinated populations.

The COVID-19 pandemic has imposed an unprecedented global health burden, with over 776 million confirmed cases and more than 7 million deaths reported worldwide as of November 2024 [1]. According to a WHO analysis, global excess mortality during 2020–2021 was estimated at 14.8 million—∼2.7 times higher than reported COVID-19 deaths—indicating substantial under-ascertainment and suggesting that excess mortality has likely persisted, reflecting the ongoing indirect effects of the pandemic [2]. In Taiwan, over 10 million confirmed cases and more than 15 000 deaths were documented by the end of November 2024 [3]. Despite widespread vaccination efforts, COVID-19 caused significant morbidity and mortality during the pandemic, particularly among vulnerable populations. In Taiwan, the 2022 Omicron surge led to a sharp increase in hospitalizations, with healthcare facilities experiencing substantial pressure due to high patient volumes, staff shortages, and the need to maintain essential non-COVID services.

Antiviral therapies, including remdesivir, molnupiravir, and nirmatrelvir–ritonavir, have played a critical role in mitigating severe outcomes [4]. The EPIC-HR trial demonstrated that nirmatrelvir–ritonavir reduced the risk of hospitalization or death by 88% compared with placebo in nonhospitalized, unvaccinated adults with laboratory-confirmed SARS-CoV-2 infection and at least one risk factor for progression to severe disease [5]. This benefit was observed when treatment was initiated within 5 days of symptom onset during a period dominated by the delta variant [5].

A meta-analysis of 32 studies demonstrated that nirmatrelvir–ritonavir significantly reduced the risks of mortality, hospitalization, and severe disease in real-world settings during the Omicron surge, with pooled relative risks ranging from 0.36 to 0.54 [6]. A more recent systematic review and meta-analysis published in 2024, which included 25 studies regarding nirmatrelvir–ritonavir, further supported the benefit of nirmatrelvir–ritonavir in reducing the risks of hospitalization, death, or their composite, with pooled relative risks ranging from 0.31 to 0.62 [7]. Although both reviews consistently suggest clinical benefit, their findings should be interpreted with caution due to heterogeneity in national prescribing guidelines and vaccination policies. These variations underscore the need for further evaluation to determine treatment effects across different subgroups. In particular, age and COVID-19 vaccination status have been explored in several observational studies as potential effect modifiers. Regarding age, several studies consistently reported treatment benefits among older adults in preventing 28- to 30-day hospitalization or death [7–12]. However, findings in younger populations have been inconsistent. Studies from the United States, Hong Kong, and Canada studies observed benefits in reducing hospitalization or all-cause mortality among younger adults [8, 9, 12], whereas others did not detect statistically significant effects in this group [10, 11, 13, 14]. Similarly, the impact of vaccination status on antiviral treatment effectiveness in preventing severe COVID-19 remains inconclusive. Studies from Colorado and Canada indicated the greatest effectiveness in preventing hospitalization and the composite outcome of hospitalization or death among individuals with 1 or 2 vaccine doses [9, 12]. In contrast, a Hong Kong study reported a reduced, though not statistically significant, risk for preventing hospitalization among fully vaccinated individuals [8]. These discrepancies highlight the need for robust, high-quality studies to generate more definitive evidence.

In Taiwan, the emergence of the omicron variant in 2022 led to a surge in cases and the rapid deployment of antiviral agents, specifically nirmatrelvir–ritonavir, which became available for COVID-19 treatment on 21 April 2022 [15]. At that time, patients were eligible to receive nirmatrelvir–ritonavir if they met the following criteria: aged 12 years or older, weighed at least 40 kg, were within 5 days of symptom onset, did not require supplemental oxygen, presented with mild to moderate illness, and had at least one specified high-risk factor for severe disease [16]. This study aims to evaluate the real-world effectiveness of nirmatrelvir–ritonavir in preventing severe COVID-19-related outcomes, including hospitalization, ICU admission, invasive ventilatory support, death, and the composite outcome of hospital admission or death, using a large, comprehensive nationwide healthcare database.

## METHODS

### Study Design and Data Sources

This nationwide, population-based retrospective observational cohort study analyzed data from the National Health Insurance Research Database (NHIRD), which captures comprehensive patient-level claims data from Taiwan's mandatory single-payer healthcare system, covering 99.9% of the 23 million Taiwanese population [17]. Data on COVID-19 vaccination status were obtained from the National Immunization Information System (NIIS), which records all government-funded vaccinations [18]. Severe COVID-19 cases and related deaths were identified through the National Infectious Disease Reporting System (NIDRS) [18], while mortality data were confirmed using the National Death Registry (NDR). The date of death was obtained from the NDR database. All case IDs required for data linkage were encrypted before being released. The linkage between all datasets is performed using a deterministic matching process based on encrypted unique personal identification numbers. Due to privacy regulations, only authorized researchers at the Health and Welfare Data Science Center are permitted to carry out this data integration [19].

### Study Population

This study was conducted in Taiwan, a country with universal health coverage and high accessibility to healthcare services. During the study period in 2022, the predominant variant of concern of COVID-19 in Taiwan was Omicron. COVID-19 diagnoses were confirmed either by polymerase chain reaction testing or by rapid antigen tests verified by medical personnel. According to the Taiwan Communicable Disease Control Act, both suspected and confirmed cases must be reported to the government within 24 hours. In line with government policy, patients who met the eligibility criteria for antiviral treatment were able to receive nirmatrelvir–ritonavir free of charge upon a physician's prescription. At least 80% of the population had received 2 doses of a COVID-19 vaccine during the study period. These healthcare and policy conditions remained stable throughout the entire study duration.

The study included patients with a confirmed diagnosis of COVID-19 (ICD-10-CM: U071 or order code: NND000) between 1 January 2022 and 1 December 2022. COVID-19 patients were excluded from the study if they met any of the following criteria: (1) aged <12 years; (2) diagnosed with COVID-19 before 1 January 2022; (3) missing information on gender; (4) had no risk factors for progression to severe COVID-19; (5) had severe kidney impairment, identified by a diagnosis of stage IV of chronic kidney disease, end-stage renal disease or by receiving dialysis, or liver impairment, identified by a diagnosis of cirrhosis, hepatocellular carcinoma or by receiving liver transplantation; (6) received molnupiravir; and (7) received nirmatrelvir–ritonavir in an inpatient setting. The date of the first diagnosis of COVID-19 was defined as the index date.

### Procedures

Drug exposure was defined as the first prescription of nirmatrelvir–ritonavir in an outpatient setting (including the emergency department) between 1 January 2022 and 1 December 2022. Patients receiving nirmatrelvir–ritonavir at the index date were categorized as the nirmatrelvir–ritonavir treated group, and patients who did not receive nirmatrelvir–ritonavir at the index date were included in the untreated group. Patients initially classified as untreated but who subsequently received nirmatrelvir–ritonavir within 30 days of diagnosis were censored at the time of treatment initiation. Data on age, gender, risk factors associated with severe COVID-19 (from the Guidelines for Clinical Management of SARS-CoV-2 Infection) [16], Elixhauser Comorbidity Index, outpatient visits in the previous year, and any hospital admission in the previous year were extracted from the NHIRD and defined as records within 1 year prior to the index date. Comorbidities were identified using ICD-10-CM codes and were considered valid if the diagnostic codes were present at least twice in outpatient clinic records or at least once during hospitalization. Vaccination status was extracted from the NIIS within 1 year prior to the index date and summarized by the number of doses received (ie, 0, 1, 2, ≥3).

### Ethics

The study received approval from the Joint Institutional Review Board of the Medical Research Ethical Foundation, Taipei, Taiwan (No. 23-S-011-1). This study was conducted and reported in accordance with the Strengthening the Reporting of Observational Studies in Epidemiology guidelines.

### Outcomes

Five outcomes were assessed, including COVID-19-related hospitalization, ICU admission, invasive ventilatory support, death, and the composite outcome of hospital admission or death. Death data were obtained from the NDR, where each case is certified by a physician. The other 4 outcomes were identified from the NHIRD, which collects data through a rigorous coding and review process. The relatedness of COVID-19 of each outcome was determined using the data from NIDRS, based on case-by-case assessments conducted through committee-led epidemiological investigations. All outcomes were evaluated within 30 days after the first COVID-19 diagnosis.

### Statistical Analysis

We calculated the incidence rate for each outcome as the number of new cases per 1000 patient-days. We used Cox proportional hazards models to estimate the adjusted hazard ratios (HRs) with 95% CIs, adjusted for age, gender, risk factors associated with severe COVID-19, Elixhauser Comorbidity, outpatient visits, any hospital admission in the previous year, and vaccination status. Patients who did not experience the outcome within 30 days following their initial COVID-19 diagnosis were right-censored at 30 days. Two sensitivity analyses were conducted to assess the robustness of the study results. First, all study outcomes were analyzed using propensity score matching with calipers of <0.2 standard deviations of the logit of propensity scores to create matched patients who shared similar observed characteristics. Second, since patients may not receive nirmatrelvir–ritonavir at the initial diagnosis, the nirmatrelvir–ritonavir was incorporated as a time-dependent variable. The degree of balance in measured covariates was assessed using the χ^2^ test for categorical variables and the Student's *t*-test for continuous variables. In addition, standardized mean differences (SMDs) were calculated to evaluate covariate balance between groups, with an SMD of <0.1 considered indicative of adequate balance. Furthermore, analyses were stratified by variables of age groups (ie, <65, ≥65) and patients aged ≥65 years with or without any risk factors, vaccination status (ie, no, yes), and doses (ie, 1–2, ≥3). All statistical analyses were conducted using SAS software, version 9.4 (SAS Inc., Cary, NC).

## RESULTS

**Figure 1.**
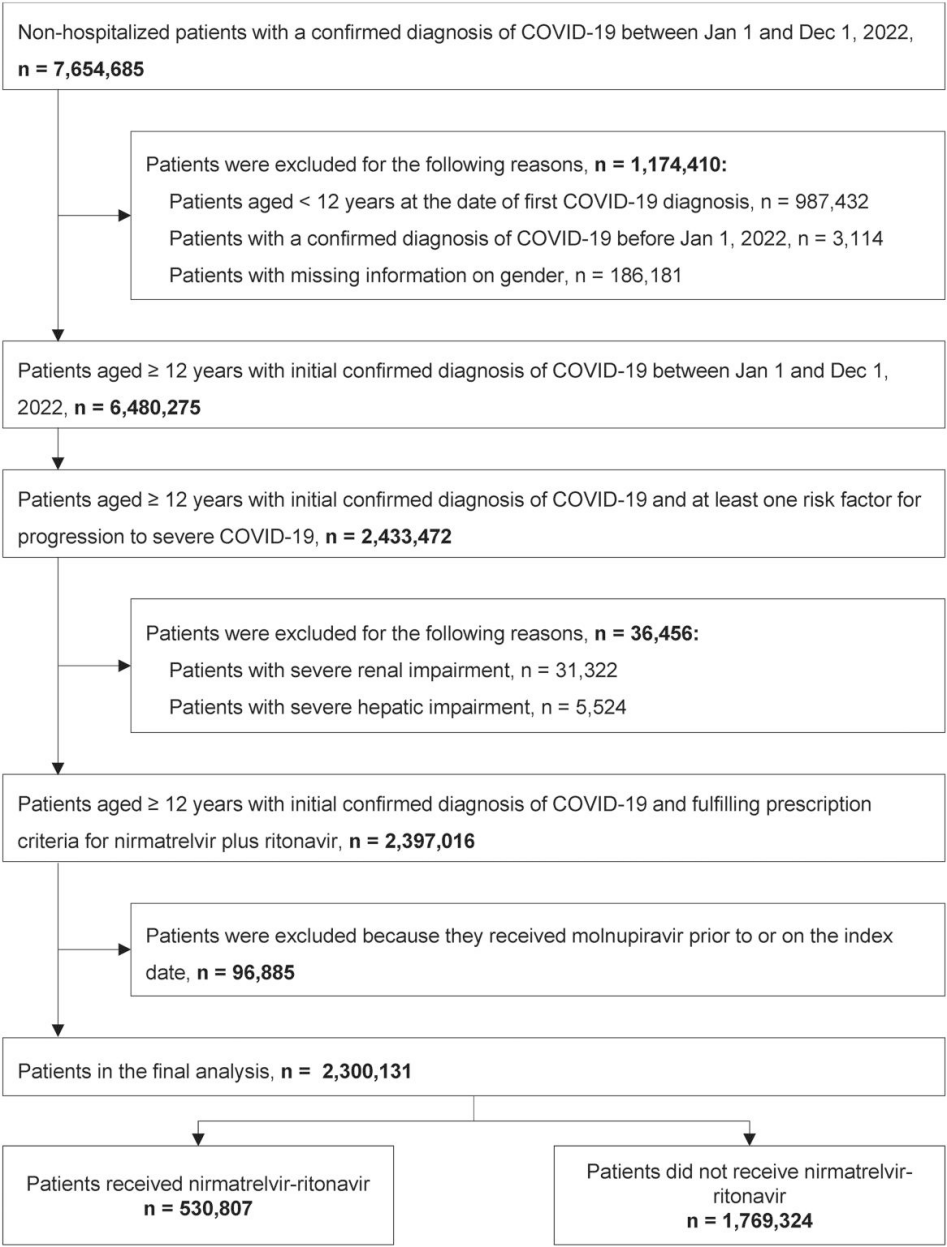
Study population flow diagram.

A total of 7 654 685 nonhospitalized patients had a confirmed diagnosis of COVID-19 between 1 January 2022 and 1 December 2022. Among these, 2 300 131 patients were eligible for analysis, comprising 530 807 patients treated with nirmatrelvir–ritonavir and 1 769 324 untreated patients (Figure 1). The majority of COVID-19 cases occurred between June and October 2022, accounting for 79.5% of the treated group (*n* = 421 950) and 69.2% of the untreated group (*n* = 1 224 020) (Table 1). The proportion of patients receiving nirmatrelvir–ritonavir increased over time, from 11.9% (57 303/482 002) between January and May to 25.6% (421 950/1 645 970) between June and October, reaching 30.0% (51 554/172 159) from November to December 2022.

**Table 1.** Characteristics of the Analysis Population

	Received Nirmatrelvir Plus Ritonavir (N = 530 807)		Did Not Receive Nirmatrelvir Plus Ritonavir (N = 1 769 324)		*P*-Value	SMD
	n	(%)	n	(%)		
Month of confirmed COVID-19 diagnosis					<.0001	
January to May, 2022	57 303	(10.8)	424 699	(24.0)	…	0.354
June to October, 2022	421 950	(79.5)	1 224 020	(69.2)	…	0.238
November to December, 2022	51 554	(9.7)	120 605	(6.8)	…	0.105
Sex					<.001	0.047
Male	234 006	(44.1)	739 280	(41.8)	…	…
Female	296 801	(55.9)	1 030 044	(58.2)	…	…
Region					<.001	
North	240 295	(45.3)	845 126	(47.8)	…	0.050
Middle	101 309	(19.1)	378 223	(21.4)	…	0.057
South	172 976	(32.6)	506 399	(28.6)	…	0.086
East	16 227	(3.1)	39 576	(2.2)	…	0.051
Age at index date (y)					<.001	
12–19	2172	(0.4)	100 388	(5.7)	…	0.310
20–29	10 904	(2.1)	272 152	(15.4)	…	0.486
30–39	20 857	(3.9)	343 520	(19.4)	…	0.497
40–49	28 622	(5.4)	316 153	(17.9)	…	0.397
50–59	36 945	(7.0)	241 637	(13.7)	…	0.222
60–64	25 540	(4.8)	116 620	(6.6)	…	0.078
65–69	146 055	(27.5)	163 430	(9.2)	…	0.486
70–74	115 238	(21.7)	100 412	(5.7)	…	0.480
75–79	58 915	(11.1)	47 570	(2.7)	…	0.337
80–84	45 487	(8.6)	35 049	(2.0)	…	0.278
≥85	40 072	(7.6)	32 393	(1.8)	…	0.273
Risk factors, *n* (%)						
Diabetes mellitus	65 355	(12.3)	89 257	(5.0)	<.001	0.260
Chronic kidney disease	14 173	(2.7)	22 449	(1.3)	<.001	0.101
Cardiovascular disease (excluding hypertension)	64 619	(12.7)	122 366	(6.9)	<.001	0.180
Chronic pulmonary disease	12 921	(2.4)	28 102	(1.6)	<.001	0.060
Immunodeficiency or immunosuppression	194 133	(36.6)	1 230 841	(69.5)	<.001	0.700
Malignancy	17 912	(3.4)	23 267	(1.3)	<.001	0.136
Tuberculosis	227	(0.0)	415	(0.0)	<.001	0.011
Chronic liver disease	6017	(1.1)	19 641	(1.1)	.153	0.002
Disabilities	2547	(0.4)	11 983	(0.7)	<.001	0.026
Mental disease	8132	(1.5)	24 899	(1.4)	<.001	0.010
Dementia	5991	(1.1)	5844	(0.3)	<.001	0.094
Asthma	6452	(1.2)	21 378	(1.2)	.672	0.001
Current and former smokers)	12 470	(2.4)	75 833	(4.3)	.195	0.108
Pregnancy and recent pregnancy (within 6 wk after childbirth)	9499	(1.8)	111 228	(6.3)	<.001	0.230
BMI ≥ 30 kg/m^2^ or >95th percentile in adolescents aged 12–17 y	4894	(0.9)	10 117	(0.6)	<.001	0.041
Elixhauser Comorbidity index					<0.001	0.172
0	334 896	(63.1)	1 258 891	(71.2)	…	…
1	195 911	(36.9)	510 433	(28.9)	…	…
Outpatient visits in previous year						
Mean (SD)	20.7 (15.84)	…	16.0 (13.56)	…	<.001	0.317
Any hospital admission in previous year					<.001	0.048
Yes	83 019	(15.6)	246 698	(13.9)	…	…
No	447 788	(84.4)	1 522 626	(86.1)	…	…
Vaccination status, *n* (%)					<.001	
0	44 634	(8.4)	83 034	(4.7)	…	0.151
1	15 714	(3.0)	53 679	(3.0)	…	0.004
2	40 619	(7.7)	195 517	(11.1)	…	0.117
≥3	429 840	(81.0)	1 437 094	(81.2)	…	0.006
Time from last vaccine dose					<.001	
No vaccine	44 634	(8.4)	83 034	(4.7)	…	0.151
0–13 d	4474	(0.8)	27 054	(1.5)	…	0.063
14–179 d	230 825	(43.5)	1 059 822	(59.9)	…	0.333
≥180 d	250 874	(47.3)	599 414	(33.9)	…	0.275

Abbreviation: SMD, standardized mean difference.

Compared with patients who did not receive the nirmatrelvir–ritonavir treatment, a higher proportion of elderly patients received nirmatrelvir–ritonavir treatment. Specifically, among treated patients, 27.5% were aged 65–69 years, 21.7% were aged 70–74 years, and 11.1% were aged 75–79 years. In contrast, the untreated group included only 9.2%, 5.7% and 2.7% in these respective age groups. Additionally, the nirmatrelvir–ritonavir treated group presented a higher percentage of diabetes mellitus (12.3%), chronic kidney disease (2.7%), cardiovascular disease (12.7%), chronic pulmonary disease (2.4%), malignancy (3.4%), mental disease (1.5%), dementia (1.1%), and BMI ≥30 kg/m^2^ for adults or >95th percentile for adolescents (0.9%), compared with the untreated group (Supplementary Table 1).

Regarding healthcare resource utilization, the treated group had more outpatient visits in the prior year (mean [SD]: 20.7 [15.84]) compared with the untreated group (16.0 [13.56]), and a higher proportion of prior hospital admissions (15.6% vs 13.9%). For the vaccination status, a higher proportion of treated patients were unvaccinated, and among the vaccinated, most had received their last dose ≥180 days prior to COVID-19 diagnosis (47.3%, *n* = 250 874) (Table 1).

Within 30 days of the index date, the nirmatrelvir–ritonavir treated group experienced 2478 (0.5%) COVID-19-related hospital admissions, 677 (0.1%) ICU admissions, 349 (0.1%) instances requiring invasive ventilatory support, 1365 (0.3%) deaths, and 2991 (0.6%) cases of either hospital admission or death. In comparison, the untreated group reported 8366 (0.5%) hospital admissions, 1923 (0.1%) ICU admissions, 1062 (0.1%) requiring ventilatory support, 3594 (0.2%) deaths, and 9479 (0.5%) combined hospital admissions or deaths. After adjusting for confounders, nirmatrelvir–ritonavir treatment was associated with significantly reduced risks for all outcomes: COVID-19 related hospital admission (HR 0.32 [95% CI .31–.34]), COVID-19-related ICU admission (0.41 [.38–.45]), COVID-19-related invasive ventilatory support (0.38 [.33–.43]), COVID-19 death (0.42 [.40–.45]) as well as hospital admission or death due to COVID-19 (0.34 [.33–.35]) (Table 2).

**Table 2.** Effectiveness of Nirmatrelvir Plus Ritonavir in Preventing Progression to Severe COVID-19-Related Outcomes Within 30 d

Outcomes	Received Nirmatrelvir Plus Ritonavir (n = 530 807)	Did Not Receive Nirmatrelvir Plus Ritonavir (n = 1 769 324)	Hazard Ratio (95% CI)^b^	*P*-Value
COVID-19-related hospital admission				
No. of cases (%)	2478 (0.5)	8366 (0.5)	…	
Incidence rate, (95% CI)^a^	0.16 (.15–.17)	0.16 (.16–.16)	0.32 (.31–.34)	<.001
COVID-19-related ICU admission				
No. of cases (%)	677 (0.1)	1923 (0.1)		
Incidence rate, (95% CI)^a^	0.04 (.04–.05)	0.04 (.03–.04)	0.41 (.38–.45)	<.001
COVID-19-related invasive ventilatory support				
No. of cases (%)	349 (0.1)	1062 (0.1)	…	
Incidence rate, (95% CI)^a^	0.02 (.02–.02)	0.02 (.02–.02)	0.38 (.33–.43)	<.001
COVID-19-related death				
No. of cases (%)	1365 (0.3)	3594 (0.2)	…	
Incidence rate, (95%CI)^a^	0.09 (.08–.09)	0.07 (.07–.07)	0.42 (.40–.45)	<.001
COVID-19-related hospital admission or death				
No. of cases (%)	2991 (0.6)	9479 (0.5)	…	
Incidence rate, (95% CI)^a^	0.19 (.19–.20)	0.18 (.18–.19)	0.34 (.33–.35)	<.001

Abbreviation: HR, hazard ratio.

^a^Events per 1000 patient-days.

^b^HRs <1 indicate that patients who received nirmatrelvir plus ritonavir were at lower risk for COVID-related outcomes compared with matched patients who did not receive nirmatrelvir plus ritonavir.

Sensitivity analyses yielded similar results, including the propensity score matching cohort (hospital admission: HR 0.31 [95% CI .30–.33]; ICU admission: 0.40 [.38–.44]; invasive ventilatory support: 0.38 [.33–.43], death: 0.42 [.39–.44]; hospital admission and death: 0.33 [.32–.34]) and the time-dependent variable in the Cox regression models (hospital admission: HR 0.31 [95% CI .29–.32]; ICU admission: 0.39 [.36–.43]; invasive ventilatory support: 0.39 [.37–.42]; death: 0.36 [.32–.41]; hospital admission and death: 0.32 [.31–.33]) (Supplementary Tables 2 and 3). Another sensitivity analysis was carried out in which the nontreatment group included only patients who never received antiviral treatment during the study period. The results are all aligned with the original analysis (Supplementary Table 4).

**Figure 2.**
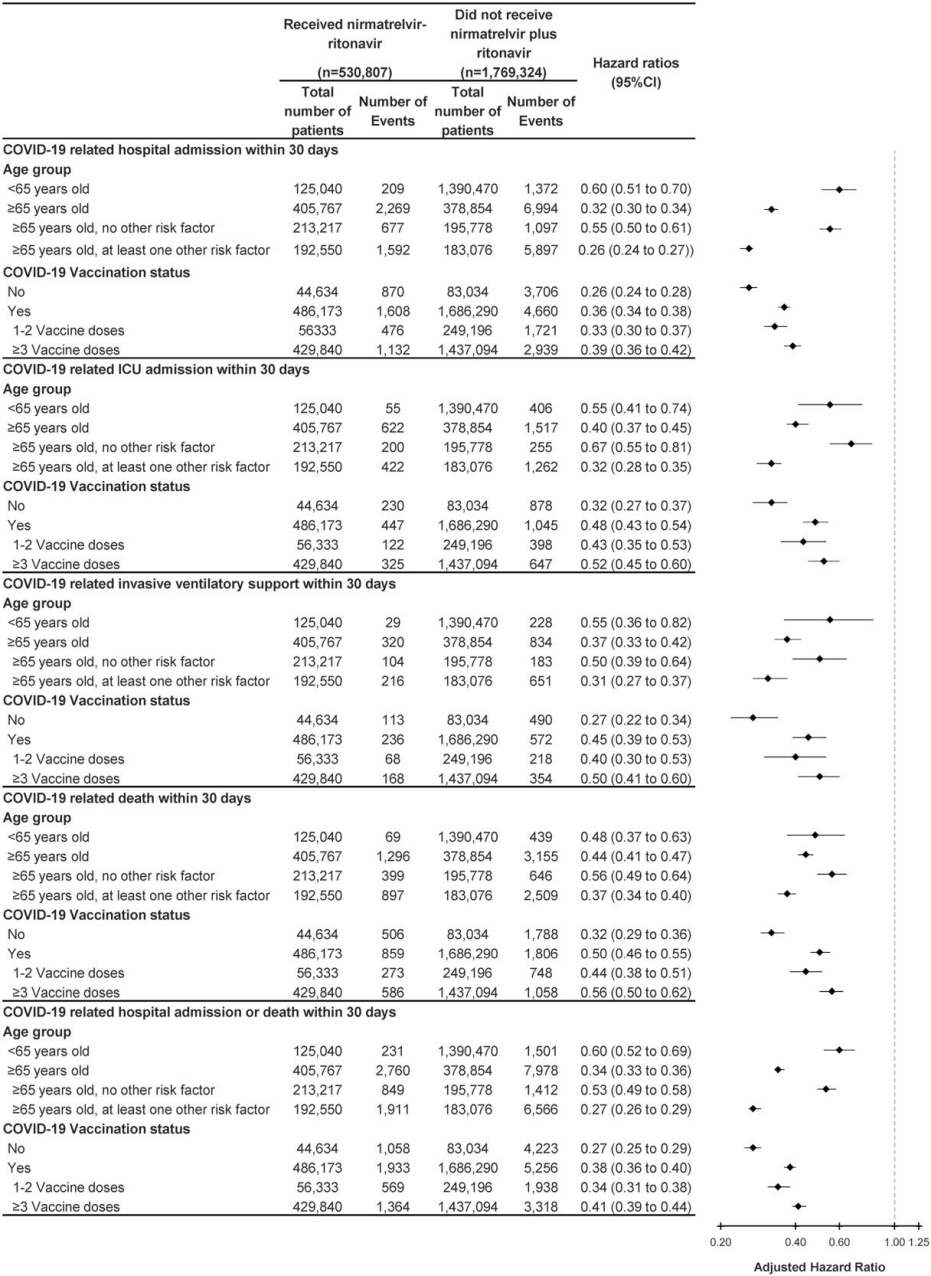
Subgroup analyses for effectiveness of nirmatrelvir–ritonavir in preventing progression to severe COVID-19 related outcomes. Multivariable Cox proportional hazards analyses were adjusted for age, gender, risk factors associated with severe COVID-19, Elixhauser Comorbidity, outpatient visits, any hospital admission in the previous year, and vaccination status. HRs <1 indicate that patients who received nirmatrelvir plus ritonavir were at lower risk for COVID-related outcomes compared with matched patients who did not receive nirmatrelvir plus ritonavir. HR, hazard ratio.

In the subgroup analysis stratified by age groups and vaccination status (Figure 2), the treated group exhibited a reduced risk of severe COVID-19-related outcomes compared with the untreated group across all subgroups. The reduction in risk for patients aged ≥65 years was slightly higher than for those aged <65 years. The effectiveness among patients aged ≥65 for each outcome was reported as follows: hospital admission (HR 0.32 [95% CI .30–.34]); ICU admission (0.40 [.37–.45]); invasive ventilatory support (0.37 [.33–.42]); death (0.44 [.41–.47]); and hospital admission or death (0.34 [.33–.36]). Notably, the treatment effect for patients aged ≥65 years with at least one additional risk factor was even more substantial. The effectiveness in this subgroup for each outcome was reported as follows: hospital admission (0.26 [.24–.27]); ICU admission (0.32 [.28–.35]); invasive ventilatory support (0.31 [0.27–0.37]); death (0.37 [.34–.40]); and hospital admission or death (0.27 [.26–.29]).

In the subgroup analysis of patients who did not receive COVID-19 vaccination (Figure 2), the reduced risk was higher than that for vaccinated patients. The effectiveness among patients without COVID-19 vaccination for each outcome was reported as follows: hospital admission (0.26 [.24–.28]); ICU admission (0.32 [.27–.37]); invasive ventilatory support (0.27 [.22–.34]); death (0.32 [.29–.36]); and hospital admission or death (0.27 [.25–.29]).

## DISCUSSION

In this large, nationwide cohort study utilizing data from Taiwan's comprehensive health insurance database, we found that nonhospitalized COVID-19 patients treated with nirmatrelvir–ritonavir had significantly lower risks of severe outcomes within 30 days postindex date compared with untreated patients. These protective effects were consistently observed across key outcomes, including COVID-19-related hospital admission, ICU admission, invasive ventilatory support, death, and the composite of hospital admission or death. Subgroup analysis stratified by age and vaccination status demonstrated a consistent trend of risk reduction across all groups, with greater effectiveness observed among patients aged ≥65 years with additional risk factors and among unvaccinated individuals. The robustness of these findings was confirmed through sensitivity analyses, including propensity score matching and time-dependent Cox models, underscoring the validity of the results.

Our study demonstrates the effectiveness of nirmatrelvir–ritonavir in reducing the risk of 5 COVID-19-related severe outcomes among patients prescribed nirmatrelvir–ritonavir in outpatient settings. Specifically, the HR for COVID-19-related hospitalization in our study was 0.32 (95% CI, .31–.34), indicating a stronger protective effect compared with studies conducted in Malaysia (HR 0.64 [95% CI, .43–.94]) [20], and Colorado (OR 0.40 [95% CI, .28–.57]) [9]. Similarly, for the composite outcome of hospitalization or death, our observed HR of 0.34 (95% CI, .33–.35) was more favorable than that reported in a Canadian cohort (OR 0.56 [95% CI, .47–.67]) [12]. Even considering death in the composite outcome from any cause, a US study reported an HR of 0.46 [21], while our study demonstrated an even higher reduced risk for nirmatrelvir–ritonavir use.

For the rest of the outcomes, death from any cause was discussed in the studies of Hong Kong (HR 0.34 [.22–.52]) [8], Canada (OR 0.49 [.40–.60]) [12], and the United States (OR 0.15 [.03–050]) [9], while we evaluated COVID-19-related death and found an HR of 0.42 [.40–.45], showing a similar effect across studies. Notably, while previous studies from Hong Kong reported no significant reduction in the risk of ICU admission or invasive mechanical ventilation with nirmatrelvir–ritonavir use [8], our study observed significant risk reductions for both outcomes: HR 0.41 (95% CI, .38–.45) for ICU admission and HR 0.38 (95% CI, .33–.43) for invasive mechanical ventilation.

In contrast, the EPIC-SR phase 2–3 trial, published in 2024, evaluated nirmatrelvir–ritonavir in nonhospitalized adults at standard risk for severe COVID-19 or fully vaccinated individuals with at least one risk factor [22]. The trial found that nirmatrelvir–ritonavir did not demonstrate a significantly difference in the COVID-19-related hospitalization or death between treatment and placebo groups. These differences may reflect variations in study populations, circulating variants, or real-world implementation [22].

During our study period, despite experiencing the Omicron variant, nirmatrelvir–ritonavir still demonstrated a significant protective effect. The stronger effectiveness observed in our study may be attributed to the high proportion of patients who received nirmatrelvir–ritonavir right on the day of diagnosis (92.1%) and within 5 days of diagnosis (99.5%). The observed effectiveness likely reflects the study context, where timely diagnosis and access to treatment were consistently ensured.

Subgroup analyses provided further insights into the differential effectiveness of nirmatrelvir–ritonavir, particularly in relation to age and vaccination status. The reduction in severe outcomes was more substantial among older adults (≥65 years), particularly those with additional risk factors, consistent with findings from a study conducted in Colorado [9]. Similarly, a US-based study demonstrated the benefit of nirmatrelvir–ritonavir across all age groups, with the greatest absolute risk reduction observed in patients aged ≥65 years [23]. Regarding vaccination status, our study found that nirmatrelvir–ritonavir was more effective in reducing severe outcomes among unvaccinated patients. This observation aligns with results from a population-based cohort study conducted in Quebec, Canada [24]. However, the treatment remained statistically significant and clinically beneficial across all outcomes in fully vaccinated individuals (≥3 doses), consistent with findings from a study in Hong Kong [8]. Notably, studies from both Colorado and Canada reported the highest effectiveness among patients who had received only 1–2 doses of a COVID-19 vaccine, compared with those who were unvaccinated or had received 3 or more doses [9, 12]. In addition, several studies conducted exclusively in vaccinated populations have further supported the effectiveness of nirmatrelvir–ritonavir in this subgroup [25, 26].

The PANORAMIC trial in the United Kingdom, which includes a nirmatrelvir–ritonavir treatment arm and enrolled vaccinated participants, is currently undergoing data cleaning and analysis, with results yet to be published [27]. Taken together, real-world evidence and randomized controlled trials continue to provide important support for the use of nirmatrelvir–ritonavir. However, the observed variability in outcomes across studies highlights the need for further investigation into how vaccination status and other host factors influence antiviral effectiveness. The potential benefits of nirmatrelvir–ritonavir warrant continued evaluation through large-scale, diverse, and methodologically rigorous studies.

Nevertheless, the subgroup analyses of this study provide valuable findings and evidence not only for the Taiwanese context but also add new insights to the broader society. Specifically, the analysis of patients aged 65 and above based on their risk factor status revealed that the protective effect of nirmatrelvir–ritonavir use was more substantial in those with at least one additional risk factor. These findings not only support clinical decision-making but also align with the approach taken by the Taiwanese government in establishing treatment eligibility criteria—drawing on published literature and tailoring policies to the characteristics of the local population to ensure a unified national treatment standard [16].

The determination of clinical outcomes is also a highlight of this study. Compared with several landmark studies from the United States, Hong Kong, and Canada, which used direct extraction of electronic medical records for the outcomes [8, 9, 12, 21], our study's outcomes were determined through committee-led investigations based on proactively reported cases to the NIDRS. These determinations accumulated into a national special topic database and the national health insurance database recording medication use. This makes our study not only the first in Taiwan but also rigorous and reliable in terms of data sources and the largest in sample size among current studies worldwide.

Despite its strengths, this study has limitations that warrant consideration. First, the NHIRD does not capture certain clinical information, such as BMI and smoking status. We relied on proxy measures (eg, diagnostic codes for obesity and smoking-related conditions), which may not fully capture these risk factors. Second, the database lacks information on the exact timing of symptom onset, preventing us from confirming whether all patients' treatment was initiated within the recommended 5-day window. This limitation could result in underestimating the drug's effectiveness, as earlier administration is known to improve outcomes. However, due to the comprehensive disease management policy in place at that time, the interval from symptom onset to diagnosis was likely very short. Third, although prescription claims confirm that patients were prescribed nirmatrelvir–ritonavir, we could not verify medication adherence or completion of the treatment course. Nonadherence may have attenuated the observed treatment effects.

## CONCLUSION

Our study demonstrates that nirmatrelvir–ritonavir significantly reduces the risk of severe COVID-19 outcomes—including hospitalization, ICU admission, mechanical ventilation, and death— among nonhospitalized patients, regardless of age and vaccination status. These findings, leveraging a large, representative population with robust data linkages and validated outcomes, provide strong real-world evidence supporting the effectiveness of nirmatrelvir–ritonavir. Future research should aim to increase the sample size and focus on high-risk subgroups to generate more precise evidence on the populations that could benefit most from the treatment.

## Supplementary Material

ofaf553_Supplementary_Data

## Supplementary Data

Supplementary materials are available at *Open Forum Infectious Diseases* online. Consisting of data provided by the authors to benefit the reader, the posted materials are not copyedited and are the sole responsibility of the authors, so questions or comments should be addressed to the corresponding author.
