# Supplementary material for: Real-World Effectiveness of Nirmatrelvir–Ritonavir Against Severe Outcomes of COVID-19 in Taiwan: A Nationwide Population-Based Cohort Study
Source: Open Forum Infect Dis. 2025 Sep 2;12(9):ofaf553. doi: 10.1093/ofid/ofaf553 (PMC12451265; doi:10.1093/ofid/ofaf553)
Supplement: ofaf553_Supplementary_Data [file ofaf553_supplementary_data.docx]

**Supplementary data to:**

Real-world effectiveness of nirmatrelvir-ritonavir against severe outcomes of COVID-19 in Taiwan: A nationwide population-based cohort study

**Supplementary Table 1.** Baseline demographics and characteristics of non-hospitalized patients before and after propensity score matching

|  | **Before propensity score matching** | | | | | | | |  | | **After propensity score matching** | | | | | | |
| --- | --- | --- | --- | --- | --- | --- | --- | --- | --- | --- | --- | --- | --- | --- | --- | --- | --- |
|  | **Received**  **nirmatrelvir plus**  **ritonavir**  **(n=530,807)** | | | **Did not receive nirmatrelvir plus**  **ritonavir**  **(n=1,769,324)** | | | **SMD**^1^ | |  | | **Received**  **Nirmatrelvir plus**  **ritonavir**  **(n=445,211)** | | | **Did not receive nirmatrelvir plus**  **ritonavir**  **(n=445,211)** | | | **SMD**^1^ |
|  | **n** | **(%)** | **n** | | **(%)** |  | |  | | **n** | | **(%)** | **n** | | **(%)** |  | |
| **Sex** |  |  |  | |  | 0.047 | |  | |  | |  |  | |  | 0.000 | |
| Male | 234,006 | (44.08) | 739,280 | | (41.78) |  | |  | | 196,785 | | (44.20) | 196,785 | | (44.20) |  | |
| Female | 296,801 | (55.92) | 1,030,044 | | (58.22) |  | |  | | 248,426 | | (55.80) | 248,426 | | (55.80) |  | |
| **Region** |  |  |  | |  |  | |  | |  | |  |  | |  |  | |
| North | 240,295 | (45.27) | 845,126 | | (47.77) | 0.050 | |  | | 194,990 | | (43.80) | 207,449 | | (46.60) | 0.056 | |
| Middle | 101,309 | (19.09) | 378,223 | | (21.38) | 0.057 | |  | | 85,977 | | (19.31) | 86,718 | | (19.48) | 0.004 | |
| South | 172,976 | (32.59) | 506,399 | | (28.62) | 0.086 | |  | | 148,938 | | (33.45) | 138,044 | | (31.01) | 0.052 | |
| East | 16,227 | (3.06) | 39,576 | | (2.24) | 0.051 | |  | | 15,306 | | (3.44) | 13,000 | | (2.92) | 0.030 | |
| **Age at index date (year)** |  |  |  | |  |  | |  | |  | |  |  | |  |  | |
| Mean (SD) | 67.45 (14.14) |  | 46.42 (18.44) | |  | 1.280 | |  | | 67.45 (14.14) | |  | 66.21 (13.53) | |  | 0.090 | |
| 12-19 | 2,172 | (0.41) | 100,388 | | (5.67) | 0.310 | |  | | 2,172 | | (0.49) | 1,938 | | (0.44) | 0.008 | |
| 20-29 | 10,904 | (2.05) | 272,152 | | (15.38) | 0.486 | |  | | 10,904 | | (2.45) | 10,344 | | (2.32) | 0.008 | |
| 30-39 | 20,857 | (3.93) | 343,520 | | (19.42) | 0.497 | |  | | 20,857 | | (4.68) | 20,249 | | (4.55) | 0.007 | |
| 40-49 | 28,622 | (5.39) | 316,153 | | (17.87) | 0.397 | |  | | 28,577 | | (6.42) | 27,615 | | (6.20) | 0.009 | |
| 50-59 | 36,945 | (6.96) | 241,637 | | (13.66) | 0.222 | |  | | 36,827 | | (8.27) | 37,685 | | (8.46) | 0.007 | |
| 60-64 | 25,540 | (4.81) | 116,620 | | (6.59) | 0.078 | |  | | 25,427 | | (5.71) | 28,986 | | (6.51) | 0.033 | |
| 65-69 | 146,055 | (27.52) | 163,430 | | (9.24) | 0.486 | |  | | 95,892 | | (21.54) | 128,265 | | (28.81) | 0.168 | |
| 70-74 | 115,238 | (21.71) | 100,412 | | (5.68) | 0.480 | |  | | 95,417 | | (21.43) | 85,855 | | (19.28) | 0.053 | |
| 75-89 | 58,915 | (11.10) | 47,570 | | (2.69) | 0.337 | |  | | 52,232 | | (11.73) | 42,489 | | (9.54) | 0.071 | |
| 80-84 | 45,487 | (8.57) | 35,049 | | (1.98) | 0.278 | |  | | 40,872 | | (9.18) | 32,122 | | (7.22) | 0.072 | |
| ≥85 | 40,072 | (7.55) | 32,393 | | (1.83) | 0.273 | |  | | 36,034 | | (8.09) | 29,663 | | (6.66) | 0.055 | |
| **Risk factors, n (%)** |  |  |  | |  |  | |  | |  | |  |  | |  |  | |
| Diabetes mellitus | 65,355 | (12.31) | 89,257 | | (5.04) | 0.260 | |  | | 63,856 | | (14.34) | 57,424 | | (12.90) | 0.042 | |
| Chronic kidney disease | 14,173 | (2.67) | 22,449 | | (1.27) | 0.101 | |  | | 13,077 | | (2.94) | 12,302 | | (2.76) | 0.010 | |
| Cardiovascular disease (excluding hypertension) | 64,619 | (12.17) | 122,366 | | (6.92) | 0.180 | |  | | 60,451 | | (13.58) | 57,715 | | (12.96) | 0.018 | |
| Chronic pulmonary disease | 12,921 | (2.43) | 28,102 | | (1.59) | 0.060 | |  | | 12,033 | | (2.70) | 11,060 | | (2.48) | 0.014 | |
| Immunodeficiency or immunosuppression | 194,133 | (36.57) | 1,230,841 | | (69.57) | 0.700 | |  | | 173,976 | | (39.08) | 170,135 | | (38.21) | 0.018 | |
| Malignancy | 17,912 | (3.37) | 23,267 | | (1.32) | 0.136 | |  | | 17,646 | | (3.96) | 14,959 | | (3.36) | 0.032 | |
| Tuberculosis | 227 | (0.04) | 415 | | (0.02) | 0.011 | |  | | 208 | | (0.05) | 201 | | (0.05) | 0.001 | |
| Chronic liver disease | 6,017 | (1.13) | 19,641 | | (1.11) | 0.002 | |  | | 5,842 | | (1.31) | 5,903 | | (1.33) | 0.001 | |
| Disabilities | 2,547 | (0.48) | 11,983 | | (0.68) | 0.026 | |  | | 2,464 | | (0.55) | 2,502 | | (0.56) | 0.001 | |
| Mental disease | 8,132 | (1.53) | 24,899 | | (1.41) | 0.010 | |  | | 7,718 | | (1.73) | 7,436 | | (1.67) | 0.005 | |
| Dementia | 5,991 | (1.13) | 5,844 | | (0.33) | 0.094 | |  | | 5,493 | | (1.23) | 4,822 | | (1.08) | 0.014 | |
| Asthma | 6,452 | (1.22) | 21,378 | | (1.21) | 0.001 | |  | | 6,138 | | (1.38) | 5,791 | | (1.30) | 0.007 | |
| Current and former smokers) | 12,470 | (2.35) | 75,833 | | (4.29) | 0.108 | |  | | 11,867 | | (2.67) | 11,958 | | (2.69) | 0.001 | |
| Pregnancy and recent pregnancy (within 6 weeks after childbirth) | 9,499 | (1.79) | 111,228 | | (6.29) | 0.230 | |  | | 9,499 | | (2.13) | 10,306 | | (2.31) | 0.012 | |
| BMI ≥ 30 kg/m2 or > 95th percentile in adolescents aged 12-17 years | 4,894 | (0.92) | 10,117 | | (0.57) | 0.041 | |  | | 4,697 | | (1.06) | 5,641 | | (1.27) | 0.020 | |
| **Elixhauser Comorbidity index** |  |  |  | |  | 0.172 | |  | |  | |  |  | |  |  | |
| 0 | 334,896 | (63.09) | 1,258,891 | | (71.15) |  | |  | | 268,155 | | (60.23) | 277,169 | | (62.26) | 0.042 | |
| 1 | 195,911 | (36.91) | 510,433 | | (28.85) |  | |  | | 177,056 | | (39.77) | 168,042 | | (37.74) |  | |
| **Outpatient visits in previous year** |  |  |  | |  |  | |  | |  | |  |  | |  |  | |
| Mean (SD) | 20.65 (15.84) |  | 15.98 (13.56) | |  | 0.317 | |  | | 21.92 (16.23) | |  | 20.83 (16.51) | |  | 0.067 | |
| **Any hospital admission in previous year** |  |  |  | |  | 0.048 | |  | |  | |  |  | |  | 0.030 | |
| Yes | 83,019 | (15.64) | 246,698 | | (13.94) |  | |  | | 78,309 | | (17.59) | 73,268 | | (16.46) |  | |
| No | 447,788 | (84.36) | 1,522,626 | | (86.06) |  | |  | | 366,902 | | (82.41) | 371,943 | | (83.54) |  | |
| **Vaccination status, n (%)** |  |  |  | |  |  | |  | |  | |  |  | |  |  | |
| 0 | 44,634 | (8.41) | 83,034 | | (4.69) | 0.151 | |  | | 40,077 | | (9.00) | 38,790 | | (8.71) | 0.010 | |
| 1 | 15,714 | (2.96) | 53,679 | | (3.03) | 0.004 | |  | | 14,443 | | (3.24) | 14,354 | | (3.22) | 0.001 | |
| 2 | 40,619 | (7.65) | 195,517 | | (11.05) | 0.117 | |  | | 37,550 | | (8.43) | 37,262 | | (8.37) | 0.002 | |
| ≥3 | 429,840 | (80.98) | 1,437,094 | | (81.22) | 0.006 | |  | | 353,141 | | (79.32) | 354,805 | | (79.69) | 0.009 | |

^1^ A difference of less than 0.1 was adopted as an indicator of good balance within the cohort

**Supplementary Table 2**. Effectiveness of nirmatrelvir plus ritonavir in preventing progression to severe COVID-19 related outcomes within 30 days after propensity score matching

| **Outcomes** | **Received nirmatrelvir plus ritonavir**  **(n=445,211)** | **Did not receive nirmatrelvir plus ritonavir**  **(n=445,211)** | **Hazard Ratio (95%CI)**^2^ | **p value** |
| --- | --- | --- | --- | --- |
| **COVID-19 related hospital admission within 30 days** |  |  |  |  |
| No. of cases (%) | 2,236 (0.50) | 6,628 (1.49) |  |  |
| Incidence rate, (95%CI)^1^ | 0.17 (0.17 to 0.18) | 0.52 (0.51 to 0.53) | 0.31 (0.30 to 0.33) | <0.001 |
| **COVID-19 related ICU admission within 30 days** |  |  |  |  |
| No. of cases (%) | 606 (0.14) | 1,488 (0.33) |  |  |
| Incidence rate, (95%CI)^1^ | 0.05 (0.04 to 0.05) | 0.11 (0.11 to 0.12) | 0.40 (0.36 to 0.44) | <0.001 |
| **COVID-19 related invasive ventilatory support within 30 days** |  |  |  |  |
| No. of cases (%) | 318 (0.07) | 809 (0.18) |  |  |
| Incidence rate, (95%CI)^1^ | 0.02 (0.02 to 0.03) | 0.06 (0.06 to 0.07) | 0.38 (0.33 to 0.43) | <0.001 |
| **COVID-19 related death within 30 days** |  |  |  |  |
| No. of cases (%) | 1,253 (0.28) | 3,013 (0.68) |  |  |
| Incidence rate, (95%CI)^1^ | 0.09 (0.09 to 0.10) | 0.23 (0.22 to 0.24) | 0.42 (0.39 to 0.44) | <0.001 |
| **COVID-19 related hospital admission or death within 30 days** |  |  |  |  |
| No. of cases (%) | 2,707 (0.61) | 7,561 (1.70) |  |  |
| Incidence rate, (95%CI)^1^ | 0.21 (0.20 t 0.22) | 0.59 (0.58 to 0.61) | 0.33 (0.32 to 0.34) | <0.001 |

^1^ Events per 1,000 patient-days

^2^ HR less than 1 indicates that patients who received nirmatrelvir plus ritonavir were at lower risk for COVID-related outcomes compared with matched patients who did not receive nirmatrelvir plus ritonavir.

**Supplementary Table 3**. Effectiveness of nirmatrelvir plus ritonavir in preventing progression to severe COVID-19 related outcomes within 30 days by using time-dependent variable in Cox proportional hazard ratio models

| **Outcomes** | **Hazard Ratio (95%CI)**^1^ | **p value** |
| --- | --- | --- |
| **Hospital admission** | 0.31 (0.29 to 0.32) | <0.001 |
| **ICU admission** | 0.39 (0.36 to 0.43) | <0.001 |
| **Invasive ventilatory support** | 0.39 (0.37 to 0.42) | <0.001 |
| **Death** | 0.36 (0.32 to 0.41) | <0.001 |
| **Hospital admission or death** | 0.32 (0.31 to 0.33) | <0.001 |

^1^ HR less than 1 indicates that patients who received nirmatrelvir plus ritonavir were at lower risk for COVID-related outcomes compared with matched patients who did not receive nirmatrelvir plus ritonavir.

**Supplementary Table 4**. Effectiveness of receiving nirmatrelvir plus ritonavir on the day of diagnosis in preventing progression to severe COVID-19 related outcomes within 30 days

| **Outcomes** | **Hazard Ratio (95%CI)**^1^ | **p value** |
| --- | --- | --- |
| **Hospital admission** | 0.30 (0.28 to 0.31) | <0.001 |
| **ICU admission** | 0.38 (0.35 to 0.42) | <0.001 |
| **Invasive ventilatory support** | 0.35 (0.31 to 0.40) | <0.001 |
| **Death** | 0.39 (0.36 to 0.42) | <0.001 |
| **Hospital admission or death** | 0.31 (0.30 to 0.32) | <0.001 |

^1^ HR less than 1 indicates that patients who received nirmatrelvir plus ritonavir were at lower risk for COVID-related outcomes compared with matched patients who did not receive nirmatrelvir plus ritonavir.

^2^ Treatment group: receiving nirmatrelvir plus ritonavir on the day of diagnosis; control group: never received nirmatrelvir plus ritonavir during observation.
